# Blood Pressure Variability and Baroreflex Sensitivity in Premature Newborns—An Effect of Postconceptional and Gestational Age

**DOI:** 10.3389/fped.2021.653573

**Published:** 2021-07-01

**Authors:** Kamil Javorka, Katarina Haskova, Barbora Czippelova, Mirko Zibolen, Michal Javorka

**Affiliations:** ^1^Department of Physiology, Jessenius Faculty of Medicine in Martin, Comenius University, Martin, Slovakia; ^2^Clinic of Neonatology, Jessenius Faculty of Medicine and University Hospital, Martin, Slovakia; ^3^Biomedical Centre Martin, Jessenius Faculty of Medicine, Comenius University, Martin, Slovakia

**Keywords:** preterm infants, blood pressure, blood pressure variability, baroreflex sensitivity, volume-clamp photoplethysmography

## Abstract

**Introduction:** Cardiovascular system is the vitally important system in the dynamical adaptation process of the newborns to the extrauterine environment. To reliably detect immaturity in the given organ system, it is crucial to study the development of the organ functions in relation to maturation process.

**Objectives:** The objective was to determine the changes in the spontaneous short-term blood pressure variability (BPV) and baroreflex sensitivity (BRS) reflecting various aspects of cardiovascular control during the process of maturation in preterm babies and to separate effects of gestational age and postnatal age.

**Methods:** Thirty-three prematurely born infants without any signs of cardio-respiratory disorders (gestational age: 31.8, range: 27–36 weeks; birth weight: 1,704, range: 820–2,730 grams) were enrolled. Continuous peripheral blood pressure signal was obtained by non-invasive volume-clamp photoplethysmography method during supine rest. The recordings of 250 continuous beat-to-beat blood pressure values were processed by spectral analysis of BPV (assessed measures: total power, low frequency and high frequency powers of systolic BPV) and BRS calculation. For each infant we also assessed systolic, diastolic and mean blood pressures, heart rate and respiratory rate.

**Results:** With the postconceptional age, BPV measures decreased (for total power: Spearman correlation coefficient r_s_ = −0.345, *P* = 0.049; for low frequency power: r_s_ = −0.365, *P* = 0.037; for high frequency power r_s_ = −0.349; *P* = 0.046); and BRS increased significantly (r_s_ = 0.448, *P* = 0.009). The further analysis demonstrated that these effects were more attributable to gestational age than to postnatal age. BRS correlated negatively with BPV magnitude (r_s_ = −0.479 to −0.592, *P* = 0.001–0.005). Mean blood pressure and diastolic blood pressure increased during maturation (r_s_ = 0.517 and 0.537, *P* = 0.002 and 0.001, respectively) while heart rate and respiratory rate decreased (r_s_ = −0.366 and −0.516, *P* = 0.036 and 0.002, respectively).

**Conclusion:** We conclude that maturation process is accompanied by an increased involvement of baroreflex buffering of spontaneous short-term blood pressure oscillations. Gestational age plays a dominant role not only in BPV changes but also in BRS, mean blood pressure, diastolic blood pressure and heart rate changes.

## Introduction

Premature infants represent a specific group of individuals with different degrees of maturation. The degree of maturity/immaturity determines their prognosis, including various abnormalities in cardiovascular system (CVS) as well as subsequently increased risk of hypertension in adulthood (1–3). To reliably detect immaturity in the given system, it is crucial to study the development of the organ functions in the dynamics of time.

The immaturity of CVS and its control as one of the most vitally important physiological systems can be potentially risky for a premature newborn (4). Although an information on the maturation related changes in heart rate and heart rate variability reflecting heart rate control by autonomic nervous system are well-know (5, 6), the data focusing on another aspects of cardiovascular control – short-term blood pressure variability (BPV) and baroreflex sensitivity (BRS) – are still very rare. It is mostly caused by the methodological difficulties and unavailability associated with continuous non-invasive blood pressure recording essential for this kind of analysis (7).

Short-term BPV is a result of complex regulation of cardiovascular system, including high-pressure baroreflex, sympathetic modulation of vasculature, regulation of cardiac activity, as well as changes in venous blood return (e.g., caused by respiration) (8, 9). Knowledge of short-term BPV and BRS can potentially provide novel insight on maturation of the cardiovascular system and its control in premature infants.

The aim of this study was to study developmental changes in BPV and BRS in preterm infants. We addressed also the hypothesis that baroreflex buffers the blood pressure oscillations and therefore in the next step we focused on the relation between BPV and BRS.

## Materials and Methods

### Subjects

A total of 33 children (15 boys and 18 girls) born in the 27th−36th gestational week with a birth weight of 820–2,730 g were recruited for the study. From this basic group, one child was classified as extremely preterm (below 28 weeks of gestational age at birth) and 14 were classified as very preterm (gestational age 28–32 weeks). At the time of examination, the children had a postconceptional age (gestational + postnatal age) of 32.3–38.4 weeks. To analyze the influence of growth after birth and deviation of birth or current weight from average values for given age we found z-scores (distance from population mean expressed in standard deviations) for both birth and current weights. Growth after birth was quantified as a difference between z-scores for current and birth weights.

Criteria for inclusion of newborns included postnatal age higher than 4 days and a wrist circumference of 45–75 mm. The infants at the time of examination were without any symptoms of respiratory or cardiovascular disorders and they did not take drugs that could affect the cardiorespiratory system. Only children who were calm during the examination were included in the study. The child's motor restlessness was one of the exclusion criteria. Other exclusion criteria included respiratory or cardiovascular disorders and chromosomal abnormalities. The infants requiring phototherapy due to hyperbilirubinemia or analgosedation at the time of measurement were also excluded from our study.

The study was approved by the Ethics Committee of the Jessenius Faculty of Medicine, Comenius University in Martin (Slovakia). Written informed consent to the infant's participation in the study was given for each child by a parent or legal guardian.

### Data Acquisition

Examination of children and data recording were performed between 8 a.m. and 3 p.m, 1–2 h after feeding. The measurement conditions, including environmental, were standardized with a reduction of visual and acoustic stimuli, constant ambient temperature set according to the gestational age of the child in the incubator or on a warming mattress. During recordings the children were in the supine position.

A Portapres instrument (FMS, Netherlands) was used for non-invasive and continuous peripheral blood pressure (BP) recording. BP registration was preceded by a selection of a cuff of the appropriate size according to the circumference of the newborn's wrist. After placing the cuff on the wrist of the right hand, which was supported and kept at heart level, the child was left at rest for at least 5 min. Subsequently, we recorded resting beat-to-beat BP values during 2–5 min. Recordings of blood pressure were stored in the device memory, transferred to a computer and processed by original FMS software. During each measurement, we recorded the child's behavior and the current oxygen saturation of blood with a pulse oximeter (Nellcor Oximax N560, USA). The average value of oxygen saturation in the whole study group was 97% (range 93–100%). Respiratory rate was measured visually from chest and abdomen movements at 30 s intervals.

Each child was connected to a monitor (Carescape V100 Dinamap Critikon, USA) for intermittent determination of systemic BP using oscillometric method. Two BP values were obtained before Portapres was used, two values after measurement by Portapres. For each infant, the oscillometrically measured BP was determined as an average of these four values.

### Data Processing

From each recording, a stationary part consisting of 250 continuous beat-to-beat values of systolic BP (SBP), diastolic BP (DBP), mean BP (MBP) values and pulse intervals were found. The mean values of SBP, DBP, MBP and heart rate (HR; calculated as a reciprocal value of pulse interval) for each newborn were calculated. The spontaneous variability of SBP was analyzed by spectral analysis using fast Fourier transform (FFT) after resampling at 4 Hz and detrending. Window size was set at 256 resampled values. Total spectral power (TP) and spectral power in the low frequency band (LF: 0.04–0.15 Hz) and in the high frequency band (HF: 0.4–1.5 Hz) were calculated. Spectral bands for the neonatal population were defined according to Andriessen et al. (10, 11). While total power reflects overall blood pressure variability magnitude, blood pressure variability in high frequency band mainly reflects the mechanical effect of venous return changes associated with respiratory activity and respiratory sinus arrhythmia effect on duration of cardiac filling during diastole (9). Oscillations of the systolic blood pressure in low frequency band are associated with vascular control related to baroreflex activity and hence they are related to parasympathetic as well as to the sympathetic activity. Because the commonly calculated ratio of low- to high-frequency oscillations magnitude is considered a controversial parameter with unclear interpretation (12), its values were not calculated.

**Figure 1:**
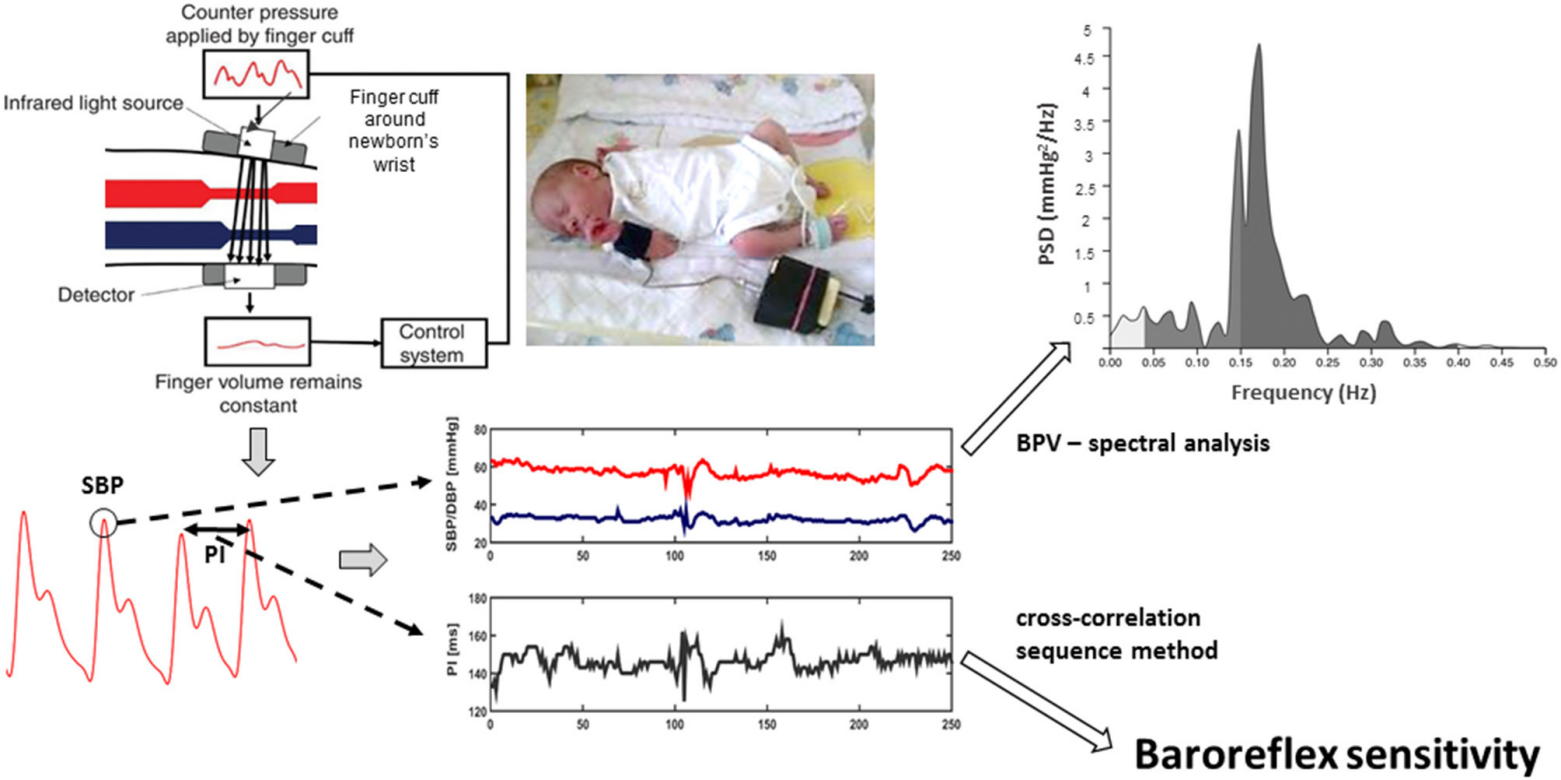
Volume-clamp photoplethysmographic method of peripheral blood pressure measurement (upper left part) is based on the application of counter pressure by cuff (in adults applied on finger, in newborns on wrist) to prevent pulsations in finger (or wrist) volume caused by blood pressure oscillations, including pulses related to heart beat. It is verified that cuff pressure then corresponds to arterial blood pressure. From the resulting recording (left bottom part of figure), systolic blood pressure (SBP) values and pulse intervals (PI) are measured. SBP oscillations (blood pressure variability) are analyzed by spectral analysis to quantify magnitude of blood pressure variability at various frequencies [e.g., in low (0.04–0.15 Hz) and high (0.4–1.5 Hz) frequency bands]. In addition, baroreflex sensitivity is quantified from the correlation between two measured signals (SBP and PI oscillations) using cross-correlation sequence method.

As the next step, BRS expressed in ms/mmHg was calculated using the cross-correlation sequence method (or xBRS method) using original software provided with Portapres device. In more details, 10 s windows of simultaneous systolic blood pressure and pulse interval changes were analyzed to find positive cross-correlation using time delays between blood pressure and pulse interval signal ranging from 0 to 5 s. When cross-correlation value was significant at *P* = 0.01, it indicated that increase in systolic blood pressure was accompanied by an increase in pulse interval pointing toward an involvement of baroreflex in given window of recording. The delay giving the highest correlation was chosen and instantaneous BRS value was estimated as the slope of relation between systolic blood pressure and pulse interval. Mean BRS for each newborn was calculated as a mean of instantaneous BRS values (average number of instantaneous BRS values used for the calculation of an individual BRS was 24). Data acquisition and processing are schematically presented in Figure 1.

### Statistical Analysis

Shapiro-Wilk normality test as a test of the normal Gaussian distribution of analyzed data was used prior to statistical analysis. Two-sample *t*-test (for variables with Gaussian distribution) and non-parametric Mann-Whitney *U*-test (for variables with non-normal distribution) were used to test significant differences between two subgroups. To determine the correlations between variables, Spearman's correlation coefficient (r_s_) and *P*-value associated with the given coefficient were determined.

For all statistics, we considered the *P*-values below 0.05 as the statistically significant. The results are presented as mean and standard deviation (SD).

## Results

The study was originally intended to analyze separately the effect of gestational age and postnatal age on BPV and BRS measures. However, infants with lower gestational age had greater instability of the respiratory and cardiovascular systems at the beginning of neonatal period. These children were examined only after the stabilization of their vital functions. It caused that children with low gestational age were usually examined later after birth (i.e., with higher postnatal age), while children with higher gestational age were examined earlier in their postnatal age. It caused strong negative correlation between gestational and postnatal age (r_s_ = −0.87) violating the possibility to analyze effects of postnatal age and gestational age separately.

Taking together, when we consider the whole group, the effect of gestational age could not be separated from the effect of postnatal age and vice versa. Therefore, we decided to correlate assessed measures with postconceptional age only combining effect of both gestational and postnatal age.

In an effort to separate effect of gestational age from the effect of postnatal age, we selected from the study group two subgroups with 11 newborns in each of them. Two subgroups of children were paired according to postconceptional age of 35 ± 1 week. To minimize the spread of postconceptional age in two groups we excluded remaining 11 infants being too far from 35th week of postconceptional age from between-groups analysis. The first group (group 1) was a group with higher gestational age and lower postnatal age and the second group (group 2) was characterized by a lower gestational age and higher postnatal age. Both groups had almost the same current weight at the time of measurement. The basic study group and subgroups characteristics are presented in Table 1.

**Table 1:** Basic characteristics including cardiorespiratory measures of the whole study group (*n* = 33) and of two subgroups with the similar postconceptional age; values are presented as mean and standard deviation (in parentheses). Range is presented in square brackets.

	**Basic study group (*n* = 33) 15 male, 18 female**	**Group 1 (*n* = 11) 6 male, 5 female**	**Group 2 (*n* = 11) 6 male, 5 female**	***P*-value**
Gestational age (weeks)	31.8 (2.7) [27–36]^*^	34.45 (1.13) [33–36]	31.73 (1.42) [29–33]^**^	<0.001^a^
Postnatal age (days)	19.4 (11) [4–39]	5.82 (1.94) [4–10]	23.82 (6.97) [15–34]	<0.001
Postconceptional age (weeks)	34.7 (1.6) [32.3–38.4]	35.27 (1.01) [33.7–36.7]	35.13 (1.11) [33.3–36.6]	0.752
Birth weight (g)	1,704 (462) [820–2,730]	2 145 (275) [1,690–2,730]	1 630 (271) [1,240–2,080]	<0.001
z-score birth weight	−0.091 (0.857) [−2.310–1.720]	−0.391 (0.848) [−2.310–0.560]	−0.179 (0.532) [−0.710–0.830]	0.492
Current weight (g)	1,983 (278) [1,260–2,700]	2 053 (280) [1,620–2,700]	2 054 (214) [1,700–2,540]	0.990
z-score current weight	−0.850 (0.789) [−3.120 to 0.430]	−1.091 (0.842) [−2.880 to 0.090]	−0.909 (0.612) [−1.520 to 0.310]	0.570
z-score difference CW-BW	−0.759 (0.293) [−1.410 to −0.140]	−0.700 (0.169) [−1.010 to −0.470]	−0.730 (0.271) [−1.170 to −0.380]	0.759
SBP (mmHg)	58.5 (5.2)	57.5 (4.0)	54.9 (4.7)	0.187
DBP (mmHg)	31.7 (7.8)	35.3 (5.5)	28.0 (5.6)	0.006
MBP (mmHg)	41.8 (6.9)	44.0 (4.8)	38.0 (5.7)	0.015
HR (min^−1^)	148.5 (14.4)	136.9 (12.0)	155.7 (9.9)	<0.001
RR (min^−1^)	50.3 (7.5)	46.9 (6.6)	51.5 (6.6)	0.113

*P-values correspond to between groups testing (Group 1 vs. Group 2). SBP, systolic blood pressure; DBP, diastolic blood pressure; MBP, mean blood pressure; HR, heart rate; RR, respiratory rate*.

* *included 14 children with gestational age in the range 28–31 weeks; 1 child with gestational age below 28 weeks*;

** *included 5 children with gestational age <32 weeks*.

a *indicates Mann-Whitney U-test used for between groups difference testing*.

BP and HR values determined by the intermittent oscillometric method in the whole basic study group did not differ significantly from the values measured by Portapres presented in Table 1. SBP determined by the oscillometric method was 58.1 (6.1) mmHg, DBP was 31.4 (8.2) mmHg, MBP was 40.6 (7.4) mmHg and HR was 149.8 (14.5 min^−1^).

### Correlations of Postconceptional Age and Z-Scores of Birth and Current Weights to Basic Cardiorespiratory Measures and to Blood Pressure Variability (BPV) and Baroreflex Sensitivity (BRS)

**Figure 2:**
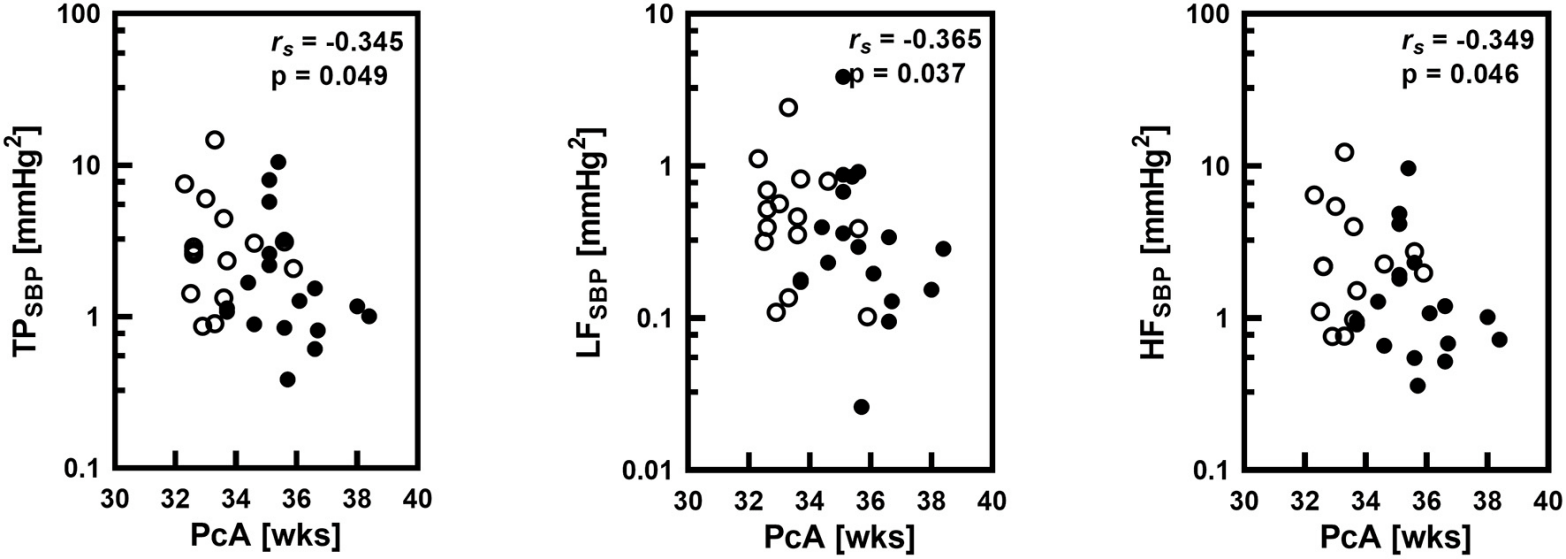
Correlations between postconceptional age (PcA) in weeks (wks) and spectral total power (TP), low frequency band spectral power (LF) and high frequency band spectral power (HF) of systolic blood pressure (SBP) variability with the Spearman correlation coefficient (r_s_) and statistical significance level (P). Logarithmic scale was applied on y-axis. Empty circles corresponds to infants with gestational age below 32 weeks.

Postconceptional age in the group of all 33 newborns positively correlated with DBP (r_s_ = 0.537; *p* = 0.001) and MBP (r_s_ = 0.517; *p* = 0.002), BRS (r_s_ = 0.448; *p* = 0.009), and it correlated negatively with HR (r_s_ = −0.366; *p* = 0.036) and respiratory rate (RR; r_s_ = −0.516; *p* = 0.002). Postconceptional age also significantly negatively correlated with total power in systolic blood pressure (TP SBP), spectral power in low frequency band (LF SBP) and in high frequency band (HF SBP) (Figure 2).

No correlation between z-scores of birth weight and current weight or of their difference with any analyzed cardiovascular measure was found.

### Effects of Gestational and Postnatal Age in Preterm Infants With Similar Postconceptional Age to Cardiorespiratory Measures and to Blood Pressure Variability (BPV) and Baroreflex Sensitivity (BRS)

Despite the similar postconceptional age and current weight, the group 1 in comparison to the group 2 had higher MBP (44.0 vs. 38.0 mmHg), DBP (35.3 vs. 28.0 mmHg) and lower HR (136.9 vs. 155.7 min^−1^).

BRS was higher and the values of the HF SBP and TP SBP were significantly lower in the group 1 with longer gestational age and shorter postnatal age in comparison to the group 2 (Tables 1, 2).

**Table 2:** Comparison between two groups of newborns (Group 1 with higher gestational age, lower postnatal age; Group 2 with lower gestational age, longer postnatal age).

	**Group 1**	**Group 2**	**P-value**
BRS (ms/mmHg)	9.42 (5.71)	3.90 (1.63)	0.010
LF SBP (mmHg^2^)	0.286 (0.251)	0.953 (1.217)	0.082
HF SBP (mmHg^2^)	1.212 (1.276)	2.727 (2.576)	0.011
TP SBP (mmHg^2^)	1.498 (1.497)	3.680 (3.232)	0.011

*Data are presented as the mean (standard deviation); P-value, significance level for between-groups comparison (Mann-Whitney U test). BRS, baroreflex sensitivity; LF SBP, spectral power in low frequency band; HF SBP, spectral power in high frequency band; TP SBP, total power in systolic blood pressure variability*.

### Baroreflex Sensitivity and Its Correlations With Blood Pressure Variability Measures and Heart Rate

**Figure 3:**
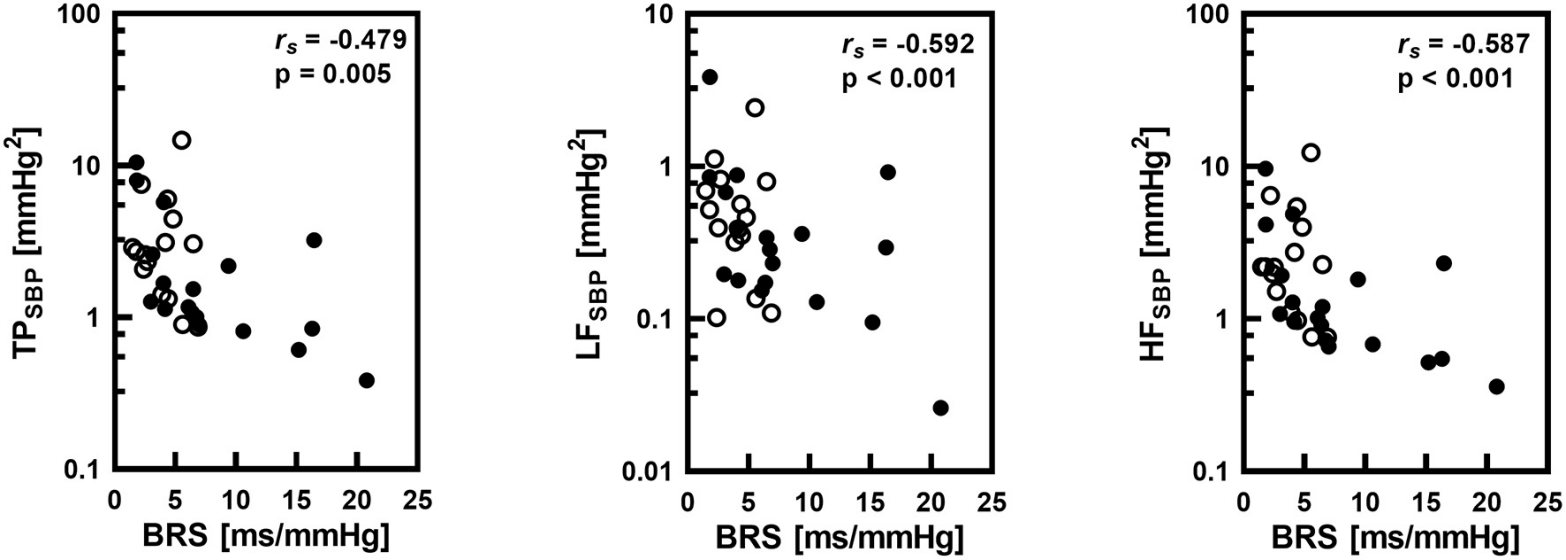
Correlations between baroreflex sensitivity (BRS) and total power (TP), low frequency band spectral power (LF) and high frequency band spectral power (HF) of systolic pressure (SBP) variability with the Spearman's correlation coefficient (r_s_) and statistical significance level (p). Logarithmic scale was applied on y-axis. Empty circles corresponds to infants with gestational age below 32 weeks.

Correlations between BRS and HR and parameters of spectral analysis of SBP variability were calculated in the whole group of 33 infants. TP as well as spectral powers in LF and HF bands significantly negatively correlated with BRS (Figure 3). There was also a significant negative correlation between BRS and HR (r_s_ = −0.594; *P* <0.001).

## Discussion

The most important observations of our study include the following effects of postconceptional age and gestational age on cardiovascular control in premature newborns: a decrease of blood pressure variabiliy accompanied by an increase in baroreflex sensitivity, a decrease in heart rate together with an increase of diastolic and mean blood pressures and a significant correlation between baroreflex sensitivity and blood pressure variability magnitude.

### Blood Pressure Variability and Baroreflex Sensitivity in Newborns

With the introduction of noninvasive method for beat-to-beat BP recording into clinical physiology, including its application to newborns, new insight into cardiovascular control emerged. Firstly, spontaneous oscillations of BP – BPV – could be analyzed providing information related to BP control. Secondly, from the analysis of heart rate (or pulse interval) changes in relation to BP perturbations, cardiac chronotropic branch of high-pressure baroreflex characteristics including BRS can be estimated.

The mechanisms behind spontaneous BPV are complex. Similarly to heart rate variability, the blood pressure changes from heart beat to heart beat. These spontaneous changes – BPV – are mostly composed of the slower (low-frequency – LF) and faster (high-frequency - HF) oscillations. Slower LF oscillations in BPV of systolic blood pressure were considered to reflect mostly sympathetic activity directed to blood vessels (13). However, mechanisms behind the LF oscillations are more complex, including other mechanisms including modulation by endothelial cells lining internal surface of vessels (9) and by spontaneous changes in smooth muscle cells in vascular wall – myogenic vascular control (8). In addition, a significant portion of LF SBP variability is attributable to spontaneous changes in heart rate – heart rate variability – affecting cardiac cycle length and hence DBP and SBP values (14).

SBP variability in the HF spectral band is mostly influenced by changes in cardiac contraction strength associated with ventilation (15). Cyclic intrathoracic pressure changes related to ventilation lead to changes in venous return and finally to fast cyclic changes in systolic blood pressure values. Similarly to LF band, venous return changes associated with heart rate variability can be also involved in the origin of HF SBP oscillations (16).

We observed the negative correlation between postconceptional age and BPV (overall BPV, as well as BPV in LF and HF bands) in the group of premature infants. It means that with a higher postconceptional age BPV in all assessed frequency bands decreases. Interestingly, when we analyzed two groups with the similar postconceptional age but different gestational and postnatal ages, we found statistically significantly lower values of TP SBP and HF SBP and a tendency toward lower LF SBP power. It indicates that the gestational age plays a dominant role in the gradual decrease of BPV in newborns.

Our results point toward the fact that the intrauterine maturation (reflected in gestational age) plays a dominant role in the stabilization of spontaneous short-term BPV, mostly in the HF band. Since blood pressure changes are mediated by multiple mechanisms partially explained above, several mechanisms could be responsible for this phenomenon:

Firstly, the decrease in the baroreflex involvement in the origin of BPV oscillations mediated by sympathetic nerves directed to the vessels can be considered. However, we assume that this mechanism will affect mostly LF oscillations in BPV and we consider this mechanism as a less probable because in our study HF SBP was affected more prominently.

Secondly, when we consider both LF and HF BPV oscillations, the changes in heart rate variability could be responsible for the changes in BPV via so called feedforward mechanism where changes in cardiac cycle length are transferred to SBP changes (16). However, based on the results of previous studies the heart rate variability increases with gestational age (5, 6) and we observed the opposite changes in BPV making this mechanism improbable.

Thirdly, the activity and regulatory role of the autonomic nervous system is underdeveloped in premature infants making mechanical changes caused by respiration dominant in the origin of BP oscillations (17). A reduction in intrathoracic pressure changes associated with breathing as a result of an increase in lung compliance and a reduction in airway resistance (18) can be responsible for the decrease in HF SBP with postconceptional and gestational age. Decreased lung compliance together with a weakening of strength of the Hering-Breuer inflation reflex (19) could be also responsible for the negative correlation between respiratory rate and postconceptional age found in our data.

Fourth important potential mechanism of observed BPV changes associated with postconceptional and gestational age include baroreflex acting as a buffer of BP oscillations (7). Wray et al. (20) found that vagal blockade reduces BRS and increases BPV in young healthy people. Therefore, we hypothesize that increased involvement of baroreflex expressed as increased BRS should be associated with a decreased BPV also in our neonatal group.

We tried to confirm this hypothesis by the analysis of BRS from spontaneous HR and SBP oscillation. In agreement with previous studies (21–24), higher BRS was present in children with higher gestational and postconceptional age. Subgroups analysis points toward a predominance of gestational age also in this relationship. We assume that a gradual decrease of BPV with postconceptional age is associated with the gradual increase of BRS. This observation is in concert with the concept of high-pressure baroreflex as a buffer of BP perturbations (25) confirming baroreflex as very important mechanism for stability of cardiovascular system even in healthy premature infants in early postnatal life. Infants with low BRS can be thus prone to circulatory instability.

### Relationship Between Postconceptional or Gestational Age and Basic Cardiorespiratory Measures

We observed a positive correlation of postconceptional age with MBP and DBP and negative correlation with heart and respiratory rate (HR, RR). In other words, the higher was the gestational age, the higher was the systemic DBP, MBP and lower HR and RR. These findings are consistent with previous results (26–29). Subgroups analysis showed that gestational age plays the dominant role in the observed changes related to postconceptional age. Interestingly, no maturation effect on SBP was observed in our study. Considering the factors influencing DBP and subsequently MBP values based on Windkessel model (30), duration of cardiac cycle (reciprocal of HR) and steepness of BP decay during diastolic phase are the most important mechanisms contributing to changes in DBP and MBP values provided that the SBP values are similar. Postconceptional age correlated negatively with HR – it would itself lead to prolongation of diastole and a decrease of DBP (and MBP) values. However, since the positive correlation was observed between DBP or MBP values and postconceptional age, it indirectly indicates a marked increase in the steepness of BP diastolic decay. It was most probably caused by an increased peripheral vascular resistance in relation to increasing postconceptional and gestational age – but this indirect finding should be confirmed by direct measurement.

Several previous studies demonstrated that regardless of gestation week at birth where marked difference in BP values are observed, BP values become similar at the postconceptional age of 42 – 44 weeks (31, 32). In agreement with previous data, the newborns from our study born more prematurely, despite having a longer postnatal age (almost 24 vs. 6 days) at the time of measurement, have not yet been able to reach BP and HR values of babies with higher gestational and lower postnatal age at the 35th postconceptional week. It clearly indicates that the duration of postnatal age alone in estimating the maturity of the cardiovascular system can be misleading when the gestational age is too low. We confirmed that postconceptional age in preterm infants is more accurate parameter predicting value of blood pressure than postnatal age.

In contrast, growth rate expressed as a difference in z-scores between birth and current weights did not correlate with any analyzed measure and it did not differ between two groups. It indicates that potential effect of altered growth rate after birth did not contribute to the observed changes and relations.

### Methodological Limitations

Method of the continuous non-invasive recordings of blood pressure by volume-clamp method in neonatology has great benefits but is relatively difficult and limited. The volume-clamp photoplethysmographic method (33) enables to record each beat-to-beat value of SBP, MBP and DBP for analysis of spontaneous BPV together with a calculation of BRS. In newborns, the reliablity of modified method (finger cuff used in adults is applied on the wrist of newborn) for determining BP and BPV values was verified and validated by comparison with the oscillometric method as well as with the invasive intraarterial blood pressure measurement (10, 34, 35).

The first limit stems from the size of the cuffs supplied with the device (Portapres, Finometer). It limits the use of the device to newborns with a wrist circumference in the range of 45–75 mm, i.e., mostly to the premature newborns. A wrist circumference in our group of premature infants was in the range 49.5–74.2 mm. Using an inappropriate cuff size (e.g., smaller cuff size for a larger wrist in the full-term newborn could result in unprecise BP readings and increased discomfort of the child. Cuff-size limitation currently restricts blood pressure variability and baroreflex analysis to premature infants – therefore it was not possible to compare premature newborns with a control group of full-term neonates to better characterize changes related to maturation.

To obtain continuous beat-to-beat recordings of systemic blood pressure in preterm infants at rest it is necessary to provide a suitable quiet and thermoneutral environment. Restlessness of a newborn, especially motoric, not only leads to physiological changes in cardiorespiratory parameters, but it can also cause artifacts by mechanical cuff compression. It could make the recording full of artifacts and hence unusable for evaluation of the spontaneous short-term BPV. In addition, although it would be interesting to observe changes in blood pressure control related to early postnatal period, due to ethical reasons the infants were examined only after stabilization of vital functions requiring ≥4 days in our study group.

Regarding the measurement length, long-term inflation of the cuff on the wrist can lead to acral venous congestion and discomfort of the child. Some authors used a measurement time interval of 4–10 min in their studies [e.g. (10, 36, 37)]. Yiallorou et al. (38) used repeated 2-min episodes to register BP in premature infants. We used a measurement time of 5 min providing sufficiently long stationary time periods (containing 250 continuous beats) for data analysis.

Relatively small number of examined subjects associated with complex measurement procedure did not enable to account for potential sex differences and it must be also considered as a study limitation.

## Conclusions

We conclude that maturation process is accompanied by a decrease in BPV and an increase of BRS reflecting buffering of BP changes by baroreflex. Gestational age plays a dominant role not only in BPV changes but also in BRS, MBP, DBP and HR changes. The results indicate changes related to parasympathetic heart rate control in relation to postconceptional and gestational age in preterm infants.

## Data Availability Statement

The raw data supporting the conclusions of this article will be made available by the authors, without undue reservation.

## Ethics Statement

The studies involving human participants were reviewed and approved by Ethical Committee of the Jessenius Faculty of Medicine, Comenius University, Martin, Slovakia. Written informed consent to participate in this study was provided by the participants' legal guardian/next of kin.

## Author Contributions

KJ, MZ, and MJ designed the study. KH and MZ arranged for the probands participation. KH performed the measurements. BC and KJ analyzed the data and prepared figures. KJ and MJ wrote the manuscript and have contributed equally to this paper and share first authorship. KJ, MJ and MZ contributed to the interpretation of the results. KJ and MZ helped to supervise the project. All authors contributed to the article and approved the submitted version.

## Conflict of Interest

The authors declare that the research was conducted in the absence of any commercial or financial relationships that could be construed as a potential conflict of interest.
